# Host attraction and biting behaviour of *Anopheles* mosquitoes in South Halmahera, Indonesia

**DOI:** 10.1186/s12936-017-1950-5

**Published:** 2017-08-02

**Authors:** Brandyce St. Laurent, Timothy A. Burton, Siti Zubaidah, Helen C. Miller, Puji B. Asih, Amirullah Baharuddin, Sully Kosasih, Shinta, Saya Firman, William A. Hawley, Thomas R. Burkot, Din Syafruddin, Supratman Sukowati, Frank H. Collins, Neil F. Lobo

**Affiliations:** 1 Eck Institute for Global Health, University of Notre Dame, Notre Dame, IN USA; 2 Eijkman Institute for Molecular Biology, Jakarta, Indonesia; 3 Centers for Disease Control and Prevention, Atlanta, GA USA; 4 Unicef, Jakarta, Indonesia; 5 National Institute of Health Research and Development, Jakarta, Indonesia; 6 Laboratory of Malaria and Vector Research, National Institute of Allergy and Infectious Diseases, National Institutes of Health, Bethesda, MD USA; 7 Australian Institute of Tropical Health and Medicine, James Cook University, Cairns, Australia

**Keywords:** *Anopheles*, Indonesia, Malaria, Biting behavior, Host attraction, Vector ecology

## Abstract

**Background:**

Indonesia is home to a variety of malaria vectors whose specific bionomic traits remain largely uncharacterized. Species-specific behaviours, such as host feeding preferences, impact the dynamics of malaria transmission and the effectiveness of vector control interventions.

**Methods:**

To examine species-specific host attraction and feeding behaviours, a Latin square design was used to compare *Anopheles* mosquitoes attracted to human, cow, and goat-baited tents. *Anopheles* mosquitoes were collected hourly from the inside walls of each baited tent. Species were morphologically and then molecularly identified using rDNA ITS2 sequences. The head and thorax of individual specimens were analysed for *Plasmodium* DNA using PCR. Bloodmeals were identified using a multiplex PCR.

**Results:**

A total of 1024, 137, and 74 *Anopheles* were collected over 12 nights in cow, goat, and human-baited tents, respectively. The species were identified as *Anopheles kochi*, *Anopheles farauti* s.s., *Anopheles hackeri*, *Anopheles hinesorum*, *Anopheles indefinitus*, *Anopheles punctulatus*, *Anopheles tessellatus*, *Anopheles vagus*, and *Anopheles vanus*, many of which are known to transmit human malaria. Molecular analysis of blood meals revealed a high level of feeding on multiple host species in a single night. *Anopheles kochi*, *An. indefinitus*, and *An. vanus* were infected with *Plasmodium vivax* at rates comparable to primary malaria vectors.

**Conclusions:**

The species distributions of *Anopheles* mosquitoes attracted to human, goat, and cow hosts were similar. Eight of nine sporozoite positive samples were captured with animal-baited traps, indicating that even predominantly zoophilic mosquitoes may be contributing to malaria transmission. Multiple host feeding and flexibility in blood feeding behaviour have important implications for malaria transmission, malaria control, and the effectiveness of intervention and monitoring methods, particularly those that target human-feeding vectors.

**Electronic supplementary material:**

The online version of this article (doi:10.1186/s12936-017-1950-5) contains supplementary material, which is available to authorized users.

## Background

Indonesia is a large and geographically diverse country with more than 30 *Anopheles* species. Malaria prevalence varies among climates and locales, with an estimated 25% of the human population residing in malaria endemic areas and an estimated 1.3 million cases of malaria in 2015 [1, 2]. Many of the resident *Anopheles* species are members of cryptic species complexes whose distribution and ecology are not well known [3, 4]. Characterizing the malaria vector species composition in Indonesia is critical to determine suitable methods for collection and the potential impact of interventions that exploit vector feeding behaviours. Multiple host feeding and flexibility in feeding behaviour may enable species to bypass the most common malaria control interventions, such as insecticide treated nets (ITNs) or indoor residual spray (IRS) which are centered around indoor and nighttime biting anthropophilic *Anopheles* [5]. Generalist host feeding behaviours can also contribute to the spread of viral zoonotic diseases of which humans are incidental hosts, such as West Nile Virus and Japanese encephalitis. Species-specific host attraction, biting preference, and the densities of available hosts may be important for the contribution of any one *Anopheles* species to local malaria transmission [6, 7]. Additionally, species that are opportunistic in host selection may be deterred from biting humans if adequate numbers of other hosts are available, with a consequent effect on reducing malaria transmission [8, 9].

Host choice experiments can help describe the relative attractiveness of different host species to mosquitoes. Host feeding is a complex behaviour and is likely determined by a combination of both innate host preferences and ecological factors, such as host seeking behaviour [10, 11] and host availability, including defensive behaviour [12–14]. The rate of human biting is a critical component of calculating vectorial capacity, an important epidemiological measure of how much a particular vector is capable of contributing to malaria transmission [15]. Human biting rates are most frequently measured using human landing collections or other types of human-baited traps [16–19], and human preference is evaluated by testing the relative attraction of humans to another animal by comparing capture rates in paired tent traps [20, 21]. Feeding success rate is measured by comparing the sources of bloodmeals in captured mosquitoes [22–25]. These measures and other host and local factors are useful in understanding mosquito feeding preferences and behaviour [26].

The bionomics of *Anopheles* species that act as secondary vectors and sustain malaria transmission become increasingly important when considering malaria elimination strategies. In addition to prompt diagnosis and treatment of human infections, it is clear that for outdoor biting, zoophilic vectors, like most members of the *Anopheles punctulatus* complex, there is a need for alternative methods of malaria control [27]. One potential approach to target mosquitoes that feed on non-human hosts is the use of endo-insecticides such as ivermectin [28, 29]. Ivermectin has been shown to reduce populations of some vectors in the south Pacific region, and may be more effective against zoophilic vectors [30]. Other methods of zooprophylaxis have been evaluated in Indonesia and shown to have some potential for control [8]. If *Anopheles* populations frequently feed on non-human hosts, interventions focused on or near cattle or other animals may be effective for reducing the contribution of these typically zoophagic or generalist feeding species to malaria transmission [31]. To establish which control measures would be most effective in an environment with multiple vectors, species-specific feeding behaviours must be clearly defined. In this study the feeding behaviours of *Anopheles* species were assessed by evaluating their attraction to cow, goat, and human hosts. If the sub-populations of *Anopheles* transmitting malaria also bite animals, animal-baited traps could be a useful and cost effective tool for monitoring vector populations.

## Methods

### Site description

This study was conducted in Saketa village, South Halmahera, in the northern Maluku islands of Indonesia. Villages are primarily coastal with houses constructed of wood and plaster with corrugated metal roofs and open eves which could provide access for mosquito entry, though indoor catches in this area have indicated limited house entering (N. Lobo, unpublished data). Saketa village is a fishing village located on the coast adjacent to heavily forested hills and has a population of roughly 5000 people. Grazing animals were held in large open fenced areas or allowed to roam freely, with an observed tendency to cluster on the periphery of the village. The primary forms of malaria mosquito control in South Halmahera are ITNs and larval habitat management. The northern Maluku islands have a rainy season from November to March. South Halmahera had some of the higher reported malaria rates in the region during the time of this study, with stable endemic transmission of *Plasmodium falciparum* and *Plasmodium vivax* [2, 32, 33].

### Study design

A Latin square design was used to compare catches of *Anopheles* attracted to human, cow, and goat-baited tents. The traps consisted of large screened tents approximately 3 m × 5 m and 2 m high (Insta-Clip, Six-sided Screen House, The Coleman Company, Inc.). One tent was used for each host. Each tent had six screened sides with two sides left completely open for mosquito entry. Humans acting as a host were protected within a small closed tent (REI “bug hut”) set inside the larger tent (Coleman 15 × 13 instant screen house). The animals in the cow and goat-baited traps were readily accessible for feeding by mosquitoes entering the trap and adult animals were used in the study. The use of host-baited tents to capture *Anopheles* mosquitoes was validated in an evaluation of sampling methods in three sites in Indonesia (MTC, unpublished), where goat-baited tents captured more *Anopheles* than either HLCs or human-baited tents. The baited tents were stationed more than 50 m apart and at least 25 m from other potential hosts. The hosts were rotated nightly through three stations for 12 nights (e.g., each host species occupied each of the 3 stations for 4 nights) in August, 2010 (during the rainy season). Resting mosquitoes were collected from the inside walls of baited tents every hour from 18:00 to 6:00 h by mouth aspiration. Human collectors were advised not to wear any insect repellants or smoke. Hourly collections were held in cups labeled by hour until processed.

### Sample processing

Specimens were morphologically identified using regional keys [34], mosquitoes were dissected for parity and abdominal status was recorded in the field. Samples were stored individually in 1.5 ml tubes and labelled with collection information and a unique identifier. This information was later paired with collection information recorded on sample forms. Samples were homogenized in a 2% CTAB (Cetyl Trimethyl Ammonium Bromide) extraction buffer, and DNA was extracted with phenol and isopropanol before precipitation with ethanol. The ribosomal DNA internal transcribed spacer region two (rDNA ITS2) region was isolated with PCR using primers developed for differentiating other *Anopheles* species complexes [35]. The ribosomal DNA internal transcribed spacer region two (ITS2) was amplified from genomic DNA using the ITS2A (5′-TGTGAACTGCAGGACACAT-3′) and ITS2B (5′-TATGCTTAAATTCAGGGGGT-3′) primers [35]. Each reaction contained 2.5 μl of 10× buffer, 200 μM of each dNTP, 0.5 units of Taq DNA polymerase, 0.75 μl of 10 pmol/μl of forward and reverse primers, and 2 μl of the prepared DNA template. The thermocycling conditions were as follows: 94 °C for 5 min, 30 cycles of denaturation at 94 °C for 1 min, annealing at 52°C for 1 min, and extension at 72 °C for 2 min, with a final extension at 72 °C for 5 min.

The presence of amplified fragments was confirmed by electrophoresis on a 1% agarose gel. An enzyme cleanup was used to purify each PCR product: 2 U of Exonuclease 1 (USB Corporation, Cleveland, OH, USA), 1 U of Shrimp Alkaline Phosphatase (USB), and 1.8 μl of ddH20 were added to 8 μl of PCR product. This mixture was incubated at 37 °C for 15 min, followed by 15 min at 80 °C to inactivate the enzymes. Purified PCR products were sequenced directly using Sanger sequencing on ABI 3730 xl DNA Analyzer platform (Applied Biosystems).

### Blood meal typing and screening for *Plasmodium*

Blood meals of visibly blood-fed specimens were analysed using a multiplex blood meal diagnostic PCR assay based on vertebrate mitochondrial DNA sequences [22] using cow, human, and goat-specific primers. Blood meals that did not match these species were sequenced using the universal forward and reverse primers of this PCR assay to identify the host blood meal source. A subset of 350 specimens was analysed for *Plasmodium* infection using the standard CDC sandwich ELISA test for the detection of *P. falciparum*, *P. vivax*-210, and *P. vivax*-247 circumsporozoite (CS) proteins [36], and a confirmatory multiplex PCR for *P. falciparum* and *P. vivax* [37].

### Analysis

Sequences were aligned using Seqman assembler (Lasergene v 8.1.5) to evaluate genetic variation, and identical sequences were grouped into contigs. The consensus sequences of these contigs were blasted against the NCBI (National Center for Biotechnology Information) database with BLASTn for confirmation of molecular species identification. Statistical analyses were performed using R version 3.3.1 (Vienna, Austria) [38]. Figures were generated using the ggplot2 and RColorBrewer packages within R version 3.3.1 [38–40].

## Results

*Anopheles* mosquitoes of nine morphologically identified species were captured (n = 1235) during 12 catch-nights in August 2010 in Saketa village, Halmahera. These morphological species were *Anopheles farauti, Anopheles hackeri, Anopheles indefinitus, Anopheles kochi, Anopheles punctulatus, Anopheles subpictus, Anopheles tessellatus, Anopheles vagus,* and *Anopheles vanus;* many of which are considered vectors of human *Plasmodium* [3, 41]. Ribosomal ITS2 sequence analysis of specimens revealed a low concordance between molecular and morphological identifications. Of 1014 specimens that were molecularly identified, 354 (34.9%) were discordant from the morphological identification (Table 1). Molecularly identified species were *An. kochi* (321), *An. farauti* s.s. (1), *An. hackeri* (1), *An. hinesorum* (18), *An. indefinitus* (112), *An. punctulatus* (2), *An. tessellatus* (4), *An. vagus* (514), and *An. vanus* (41). Five of these species were found in all three host-baited tents in similar proportions (Table 2). Morphologically identified *An. hackeri* and *An. vanus* had not been previously sequenced. These sequences have been submitted to GenBank. ITS2 sequences within each molecular species were identical, and hereafter, the “species” name will refer to molecularly identified species. Five of nine molecular species (eight of nine by morphological ID) were captured on each of the three hosts (Table 2).

**Table 1:** Morphological ID (columns) versus molecular ID (rows)

	*An. farauti*	*An. hackeri*	*An. indefinitus*	*An. kochi*	*An. punctulatus*	*An. tessellatus*	*An. vagus*	*An. vanus*	*An. subpictus*	Male
Molecular species ID										
*An. farauti* s.s					1					
*(An. hackeri)*		*1*								
*An. indefinitus*			*87*	3	1		1		19	1
*An. kochi*			9	*309*		2	2			
*An. punctulatus*					*2*					
*An. tessellatus*						*4*				
*An. vagus*	1		283	7			*213*	4		6
*(An. vanus)*								*40*		
*An. hinesorum*	*4*			1		13				
Unknown					1	4	1			
Not amplified	1	0	152	9	0	1	11	39	0	2

Species in (parenthesis) have not had their ITS2 region previously sequenced. Italics indicated concordance of identification to morphological group

**Table 2:** Molecular species identification of mosquitoes caught in three host-baited traps

Molecular identification	Host baited tent			
	Cow	Goat	Human	Total
*(An. hackeri)*	1			1
*(An. vanus)*	32	6	2	40
*An. farauti* s.s.		1		1
*An. hinesorum*	14	2	2	18
*An. indefinitus*	99	10	3	112
*An. kochi*	267	42	13	322
*An. punctulatus*			2	2
*An. tessellatus*	4			4
*An. vagus*	448	38	28	514
Unknown	4	1	1	6
Total	869	100	51	1020

Total molecularly confirmed specimens in each host-baited tent are n = 865 in the cow-baited tent, n = 99 in the goat-baited tent, and n = 50 in the human tent

Blood meal analysis revealed that *Anopheles* of three of the nine species, *An. indefinitus*, *An. kochi*, and *An. vagus,* had fed on all 3 host species (Additional file 1: Table S1). 8.6% (93 of 1088) of blood-fed *Anopheles* contained blood from more than one host species (Table 3). 19.7% of blood meals (n = 214) contained blood from a host other than the host in the tent where the mosquito was captured. The percentage of human-fed females was higher in the human and goat-baited tents, 11.5 and 7.4% respectively, with a much lower proportion of human-fed females in the cow-baited tent, 1.1% (Table 3). Counting mixed blood meals as two separate blood meals, blood feeding rates of total *Anopheles* analysed were: human 2%, goat 14%, and cow 84% (Table 3). Human blood index could not be calculated from this data as the relative numbers of cows, goats, and humans in the village were not tallied.

**Table 3:** Bloodmeal identification of molecularly confirmed species

	Host baited tent			
	Human	Goat	Cow	Total
Single blood meal				
Human	*2*	*3*	*2*	*7*
Goat	12	38	38	88
Cow	29	37	834	900
Mixed blood meals				
Cow + goat	5	13	59	77
Cow + human	*3*	*3*	*8*	*14*
Goat + human	*1*	*1*	*0*	*2*
Total	52	95	941	1088
Human fed (%)	*11.5*	*7.4*	*1.1*	*2.1%*
Mixed bloodmeals (%)	17.3	17.9	7.1	*8.5%*

Rates of human and mixed bloodmeals as percentages of total per host tent are shown. Human containing bloodmeals are in italics

**Fig. 1:**
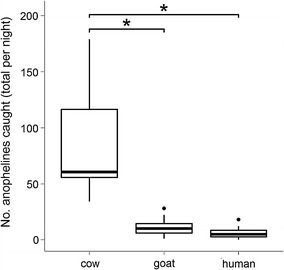
Number of mosquitoes caught per trap over all nights. Total specimens caught in each host-baited tent are n = 1024 in the cow-baited tent, n = 137 in the goat-baited tent, and n = 74 in the human tent

The total *Anopheles* catch for each host-baited tent was compared using ANOVA and a post hoc Tukey’s HSD test. The cow-baited tent caught significantly more *Anopheles* (n = 1024) per night than either the goat or human baited tents (Fig. 1; *p* < 0.001). *Anopheles* numbers captured in the human and goat-baited tents were not significantly different from each other, with 74 and 137 *Anopheles*, respectively. Numbers of *Anopheles* were not significantly affected by location (*p* = 0.325) or night of capture (*p* = 0.470).

**Fig. 2:**
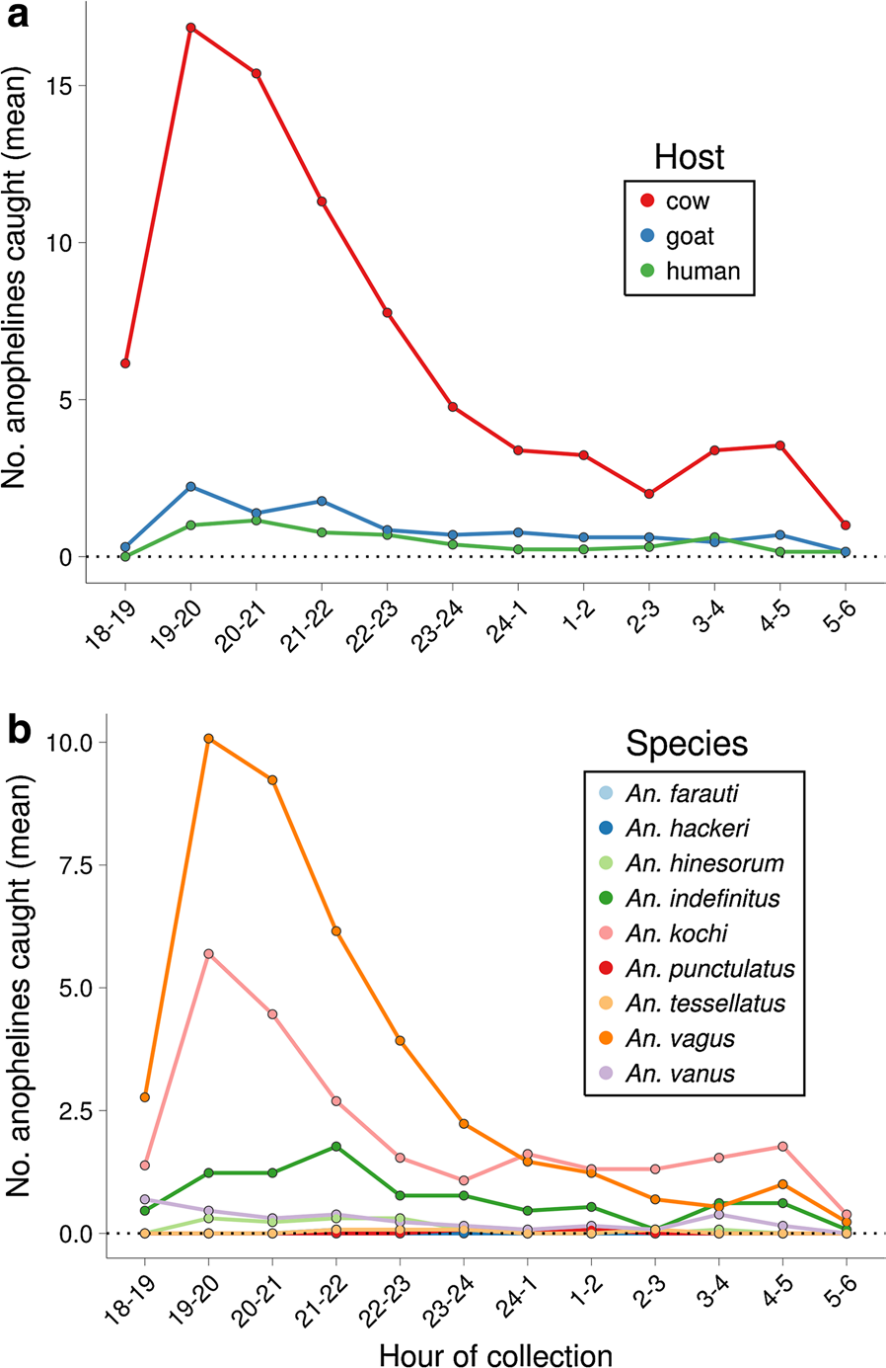
Hourly biting behaviour by host bait and species. **a** Total *Anopheles* by hour by host baited tent. **b** Total number of molecular species by hour. For most species, an early evening peak of activity was observed around 7–8 pm in all of the tents, a time when people are outdoors and active

The majority of specimens in each host-baited tent were captured before 2200 h (Fig. 2a). Entry of *An. vagus* and *An. kochi*, the two most abundant species, into the tents peaked from 1900 to 2100 (Fig. 2b). Nine male *Anopheles* were collected, 6 in the cow-baited tent, two in the goat-baited tent, and one in the human-baited tent. Six of the males were molecularly identified as *An. vagus*, and one was *An. indefinitus*, with two unknowns.

A subset of 350 specimens were analysed using a sandwich ELISA for CS protein [36] and by PCR for *Plasmodium* DNA [37]. Of 336 specimens successfully analysed, 9 (2.7%) were positive for *Plasmodium vivax* DNA (Table 4) and no samples were found positive for *P. falciparum*. Six of the *P. vivax* positive specimens were identified as *An. kochi*; four collected in the cow-baited tent and two collected in the goat-baited tent. The other positive samples were *An. indefinitus* (n = 1) and *An. vagus* (n = 1) captured in the goat-baited tent, and a morphologically identified *An. barbirostris* (n = 1) captured in the human-baited tent. Six of 69 (8.95%) *An. kochi* and 1 of 10 *An. vagus* analysed were positive for *Plasmodium* DNA.

**Table 4:** Sporozoite positive mosquitoes

Molecular species ID	Host-baited tent	Blood meal ID	Positive/total	Sporozoite rate (%)
*An. indefinitus*	Goat	Goat	1/28	3.57
*An. kochi*	Goat	Unfed	6/67	8.95
	Cow	Cow		
	Cow	Cow		
	Cow	Cow		
	Cow	Cow		
	Goat	Unfed		
*An. vagus*	Goat	Cow + goat	1/206	0.49
*An. vanus*	Human	Unfed	1/10	10.0

Nine mosquito specimens of 336 analysed by CS ELISA and PCR for sporozoite antigens were found positive for *Plasmodium vivax*. None of the nine positive specimens were discordant between morphological and molecular identification

## Discussion

In a single village in South Halmahera, Indonesia, *Anopheles* mosquitoes captured in human, goat, and cow-baited tents were compared to determine species-specific host attraction and feeding behaviour. Nine *Anopheles* species were molecularly identified in these collections: *An. kochi*, *An. farauti* s.s., *An. hackeri*, *An. hinesorum*, *An. indefinitus*, *An. punctulatus*, *An. tessellatus*, *An. vagus*, and *An. vanus* (Table 1). Additionally, bloodmeals of fed mosquitoes were typed and mosquitoes were analysed for the presence of *Plasmodium* spp. sporozoites. Frequent multiple host-feeding within a single night and flexibility in host choice within *Anopheles* species were observed.

There was a large discrepancy between morphological and molecular *Anopheles* species identifications, with 35% of specimens showing discordance between morphological and molecular identifications (Table 1). This could likely be the result of the presence of many cryptic species complexes and high *Anopheles* diversity in the country. Some species may have overlapping or inconsistent morphological characteristics that confuse species identifications [42, 43]. Characterizing these traits may uncover vulnerabilities in vector populations, outside of well understood and more easily exploited anthropophilic or endophilic behaviours that may be targeted for control.

A pilot experiment, using an empty tent as a control, showed that *Anopheles* did not enter and rest on the walls of an empty tent. An experiment evaluating other human-baited trapping techniques showed that *Anopheles* would not enter tents with restricted openings, such as the Ifakara tent trap^1^ [44]. Further, few males (9 of 1235) were captured in any of the tents, indicating that the host-baited tents selectively capture females where the presence of the host was attractive, whether they were actively host-seeking or had just fed. 24% of female *Anopheles* were unfed in the human-baited tent, in which the human was protected, whereas the goat or cow-baited tents contained only 18 and 5% unfed females, respectively (Table 3). Anopheline mosquitoes collected in a particular host-baited tent were expected to have fed on that host. The human hosts were protected under a smaller tent within the large tent, suggesting that the bloodfed mosquitoes captured in that tent had fed prior to entering. Mixed host blood meals might also indicate that mosquitoes entering the tents could have fed or partly fed on another host before entering the tent. High numbers of females partially fed on human hosts has been observed in other vectors, possibly due to increased disturbance when feeding on humans [21]. The increased feeding success on cow baits may contribute to greater numbers of *Anopheles* resting on inner walls of the cow-baited tents.

The species found positive for *P. vivax* sporozoites (*An. kochi, An. vagus, An. indefinitus*, and *An. vanus*) are not considered to be primary malaria vectors (Table 4). All sporozoite positive specimens were positive for *P. vivax*, consistent with studies that report that most of the malaria in this area is due to *P. vivax* [45, 46]. The majority of the *P. vivax* positive specimens were collected in the animal-baited tents. Further evaluation is needed to clarify which *Anopheles* species are acting as primary vectors and whether designations of “zoophagic” and “anthropophagic” are really applicable to the anophelines in this region in relation to monitoring and control. In this study, seemingly zoophagic vectors are feeding on both human and animal hosts and carrying *Plasmodium* parasites, potentially playing an important role in transmission.

More *Anopheles* mosquitoes were captured during the 12 nights of this experiment than by separate longitudinal sampling over the proceeding 2 years using HLCs in the same location (N. Lobo, pers. comm..), indicating zoophagic behaviours of the local vector population. Five of nine molecular species were found in all three traps (Table 2). The cow-baited tent seemed to be particularly effective for collecting *Anopheles* throughout the night and molecular analysis showed that these were the same species that were biting humans. Eight of nine *Plasmodium* positive *Anopheles* were captured in the animal-baited tents (Table 4). This result is consistent with the presence of a single population of mosquitoes in each species that shows behavioural plasticity in host species selection for blood meals, suggesting that trapping using animal baits may be an effective tool for monitoring malaria vectors. Previous evaluation of trapping techniques in this area showed that a goat-baited tent caught significantly more *Anopheles* than either a human-baited tent or human landing collections in the same village (See footnote 1). An additional variable, the effect of host size in attracting *Anopheles* mosquitoes, may be operating. Larger hosts have been shown to be more attractive to *Anopheles* [47, 48]. Mosquitoes attracted to long-range host cues such as CO_2_ and chemical volatiles may encounter more of an attractive signal from a large mammal such as a cow than a human or goat [7, 14, 49]. However, this study examined the attractiveness of single adult host-baits rather than weight or CO_2_ equivalent hosts per tent. The cow-baited tents were significantly more attractive than either the goat or human tents. Host size was likely not the only factor, since the goats used were much smaller than humans and a single goat attracted many more *Anopheles* per night than a single human host.

The majority of species found in this study (5 of 9 species) were attracted to multiple animal hosts (Table 2). Molecular identification of all *Anopheles* revealed that there were similar species distributions of mosquitoes attracted to human, goat, and cow hosts and a similar distribution of species feeding on those hosts (Table 2; Additional file 1: Table S1). Almost a fifth of the *Anopheles* captured had fed on two or more host species in the same night (Table 3; Additional file 1: Table S1). This behaviour indicates that these species may not be persistent when feeding on a host, if disturbed they may move on to another host [23]. This type of behaviour has been observed in other vectors and it has been suggested that this is due to frequent biting, which may increase human risk to malaria infectious bites or zoonotic infections [23, 52, 53].

The expected primary malaria vectors in this region would be those of the *Anopheles punctulatus* species complex, including *An. farauti* and *An. punctulatus*, both major malaria vectors from eastern Indonesia through Papua New Guinea [50, 51]. These two species represented a very small portion of the total catch in this experiment, only 3 of 1014 anophelines. The finding of a high sporozoite infection rate in *An. kochi* (9%) was surprising but may also be a function of a small sample size collected during a limited time period. Additional collections will be required to ascertain the importance of this or other species as malaria vectors in eastern Indonesia.

The peak collection of most of the species collected was early in the evening, around 19:00–20:00, when people are still very active and would likely not be protected under bed nets or indoors (Fig. 2). Though previous collections in this area also indicate that the *Anopheles* tend not to enter houses, it is even more unlikely that interventions such as bed nets or indoor residual spray would be as effective if the primary vectors are biting people early in the evening.

## Conclusions

Further population genetic analysis is needed to determine whether subpopulations of a single species are feeding on different hosts, which has implications for sampling strategies. Interventions utilizing animals could be effective in monitoring vectors or reducing malaria transmission where vectors are outdoor feeding, frequently feeding, and more zoophilic or catholic than anthropophilic, as is suggested from the blood meal analyses presented here. This study also represents a single time point in the year during the peak season of malaria transmission. Longitudinal sampling through the year in multiple locations in Indonesia would be needed to examine the feasibility of using animal-baited tents to monitor malaria vectors and to better characterize species-specific host feeding behaviours.

## Additional file

**Additional file 1: Table S1.** Bloodmeal identification of 968 mosquito abdomens by molecular species and host-baited tent. Bloodmeal source is denoted by letter: C for cow, G for goat, H for humans, U for unfed, and combination letters for multiple bloodmeals.

## Footnotes

1 Co-submitted paper: Comparative Evaluation of Anopheline Sampling Methods in Three Localities in Indonesia
