# Supplementary material for: Host attraction and biting behaviour of Anopheles mosquitoes in South Halmahera, Indonesia
Source: Malar J. 2017 Aug 2;16:310. doi: 10.1186/s12936-017-1950-5 (PMC5540179; doi:10.1186/s12936-017-1950-5)
Supplement: Supplementary file 1 — Additional file 1: Table S1. Bloodmeal identification of 968 mosquito abdomens by molecular species and host-baited tent. Bloodmeal source is denoted by letter: C for cow, G for goat, H for humans, U for unfed, and combination letters for multiple bloodmeals. [file 12936_2017_1950_MOESM1_ESM.docx]

|  | **Bloodmeal ID by Host-baited Tent** | | | | | | | | | | | | | | | | | | | | |
| --- | --- | --- | --- | --- | --- | --- | --- | --- | --- | --- | --- | --- | --- | --- | --- | --- | --- | --- | --- | --- | --- |
|  | **COW** | | | | | | | **GOAT** | | | | | | | **HUMAN** | | | | | | |
| **Molecular species:** | **C** | **G** | **H** | **U** | **CH** | **GH** | **CG** | **C** | **G** | **H** | **U** | **CH** | **GH** | **CG** | **C** | **G** | **H** | **U** | **CH** | **GH** | **CG** |
| ***An. farauti s.s.*** |  |  |  |  |  |  |  |  | **1** |  |  |  |  |  |  |  |  |  |  |  |  |
| ***An. hackeri*** |  |  |  | **1** |  |  |  |  |  |  |  |  |  |  |  |  |  |  |  |  |  |
| ***An. hinesorum*** | **7** | **3** |  |  |  |  |  |  |  |  | **2** |  |  |  |  | **2** |  |  |  |  |  |
| ***An. indefinitus*** | **78** | **3** |  | **7** | **1** |  | **6** | **4** | **3** |  | **1** |  |  |  | **2** |  |  |  |  |  |  |
| ***An. kochi*** | **234** | **7** |  | **10** |  |  | **10** | **8** | **15** | **1** | **13** |  |  | **3** | **3** | **2** | **1** | **3** |  |  | **4** |
| ***An. punctulatus*** |  |  |  |  |  |  |  |  |  |  |  |  |  |  |  |  |  | **2** |  |  |  |
| ***An. tessellatus*** | **3** |  |  |  |  |  | **1** |  |  |  |  |  |  |  |  |  |  |  |  |  |  |
| ***An. vagus*** | **370** | **21** | **2** | **15** | **4** |  | **27** | **11** | **9** | **2** |  | **3** | **1** | **4** | **9** | **6** | **1** | **5** | **3** | **1** |  |
| ***An. vanus*** | **23** | **2** |  | **2** |  |  | **1** | **2** | **1** |  |  |  |  |  | **1** |  |  | **1** |  |  |  |
| **Totals (968)** | **715** | **35** | **2** | **36** | **5** | **0** | **45** | **25** | **29** | **3** | **16** | **3** | **1** | **7** | **15** | **10** | **2** | **11** | **3** | **1** | **4** |

**Table S1 Bloodmeal identification of 968 mosquito abdomens by molecular species and host-baited tent.** Bloodmeal source is denoted by letter: C for cow, G for goat, H for humans, U for unfed, and combination letters for multiple bloodmeals.
